# Cognitive Representations (Metaphorical Conceptualizations) of PAST, FUTURE, JOY, SADNESS and HAPPINESS in Depressive and Non-depressive Subjects: Cognitive Distortions in Depression at the Level of Notion

**DOI:** 10.1007/s10936-014-9286-6

**Published:** 2014-02-09

**Authors:** Marlena Bartczak, Barbara Bokus

**Affiliations:** Faculty of Psychology, University of Warsaw, Stawki 5/7, 00-183 Warsaw, Poland

**Keywords:** Depression, Metaphor, Valence, Notions, Concepts

## Abstract

The aim of this study was to see if and how the intensity of depression correlates with the cognitive representation of notions, and if any influence is reversed during remission. The cognitive representation indices used were the valence and number of metaphors produced for a notion. Three adult groups took part: persons with depression ($n = 30$), persons in remission ($n = 12$), and a control group ($n = 30$). Five notions were considered: PAST, FUTURE, JOY, SADNESS, and HAPPINESS. The Questionnaire of the Metaphorical Conceptualization of a Notion was used. The results showed that (a) depressive subjects did not have problems with metaphorical processing, (b) depressive subjects demonstrated strong interpretational negativism, (c) subjects during remission did not present distorted conceptual processing. The results are discussed in the context of theories of automatic metaphor processing, and conceptions of cognitive depressive distortions, in tasks requiring effort and substantial involvement of cognitive resources.

## Introduction

### Cognitive Disturbances in Depression Possibly Affecting the Comprehension of Notions

Depression is a disease involving low mood and changes in emotional reactivity, as well as integral disturbances in cognitive functioning (cf. the diagnostic standards ICD-10, Pużyński and Wciórka 1997; and DSM-IV, APA 1994). The literature describes a number of disturbances of cognitive function in depression (for a brief review, see Talarowska et al. 2009), including executive dysfunction (e.g., Holmes and Pizzagalli 2007), memory deficits (including those affecting short- and long-term memory, outlined e.g., in Georgieff et al. 1998; working memory, e.g., Fossati et al. 1999; von Hecker and Sędek 1999; autobiographical memory: for a review, see Williams et al. 2007), problems with concentration (e.g., von Hecker and Meiser 2005), and problems with complex problem solving (cf. e.g., Sędek et al. 2010). Herein is a more detailed discussion on attention and memory dysfunction, both related to depression, since these aspects of cognitive functioning seem particularly important in the metaphorical processing of notions. Throughout this paper, *notions* (in the sense of individuals’ concepts) are understood as mental representations of elements of the external or inner reality. Thus notions are “concepts in use”.

Based on an overview of contemporary research on the underlying causes of cognitive deficits in depressive subjects, one could conclude that attention function is a key area of cognitive functioning, subject to distortion in connection with depression. There is evidence that people suffering from depression devote too much of their cognitive resources to processing information related to their own mistakes and depressive mood (see e.g., Stordal et al. 2004; Dalgleish et al. 2007, cited in Piotrowski and Wierzchoń 2009), or—more generally—information unimportant to the task in hand (e.g., von Hecker and Meiser 2005), making them unable to perform a cognitive task properly. Recent research results confirm the content-specificity hypothesis (Beck 1976) offered as part of Beck’s theory of depression (1963, 1967), one of the best-known, and empirically most often confirmed, theories of depression (cf. Solomon and Haaga 2005). This hypothesis states that negative stimuli (verbal or nonverbal) strongly attract the attention of depressive subjects (there are numerous examples of empirical confirmation, e.g., Blaut 2003; Lamberton and Oei 2008; for a review, see e.g., Gotlib and Neubauer 2000). Furthermore, people with depression interpret neutral stimuli as negative more often than healthy people (cf. e.g., the research of Gollan et al. 2008, on the interpretation of facial expressions by depressive individuals). One recent explanation for such a negativity bias in depression claims that it has complicated origins and is the effect of a distorted bottom-up mechanism of emotional content processing, as well as weakened top-down cognitive control over affective interference (see Fales et al. 2008, for fMRI studies using tasks involving evaluation of affectively marked stimuli). (An exhaustive overview of concepts explaining the mechanism by which serious depression influences the way attention focuses on affective stimuli can be found in Bylsma et al. 2008.)

Memory is another area of cognitive deficits in depression, well documented by empirical research. General memory impairment has even been included in the diagnostic standards for major depression proposed by the DSM-IV manual of the American Psychiatric Association (APA 1994, p. 322). Specific biases of memory in depression are also mentioned, such as better memory retention of negative information related to depression (cf. the theory of negative cognitive patterns of Beck 1976; for an overview of depression-related memory deficits, see e.g., Ellwart et al. 2003). It is considered that performance in memory tasks can be treated as a predictor of difficulties associated with emerging out of depression (in particular, this applies to remembering specific episodes from the past, see Brittlebank et al. 1993, as cited in McNally 2006). One currently prominent trend in research on distorted memory function in people with depression involves studies on working memory. The results of many empirical studies suggest the existence of working memory deficits in people suffering from depression (e.g., Fossati et al. 1999; von Hecker and Sędek 1999).

Difficulties with attention and memory function in depressive subjects are sometimes connected with ruminations (Joormann and Gotlib 2008, cf. also Levens et al. 2009), that is, automatic and uncontrolled negative thoughts about oneself, the world, and the future, typical of depression (Joormann and Gotlib 2008). One explanation for rumination states that a negative mood activates matching representations. Due to their poorer executive functions (especially inhibition), people with depression are unable to refresh representations in their working memory effectively (e.g., Friedman and Miyake 2004, as cited in Joormann and Gotlib 2008), and therefore find it difficult to replace negative representations with positive ones (that would improve their mood), thus falling into a vicious circle of ruminative thoughts (for an alternative explanation, see e.g., Levens et al. 2009; for research on the impact of rumination on cognitive functioning in depression, see e.g., Joormann and Gotlib 2008; Levens et al. 2009; Watkins and Brown 2002).

It is worth noting that the general conclusion that attention dysfunctions and memory deficits in depressive subjects make performing cognitive tasks difficult would be an oversimplification. Contemporary literature on the cognitive functioning of depressive subjects includes numerous reports on cognitive tasks performed by this group, but the results of meta-analyses suggest that the results of these studies are often inconsistent. von Hecker and Sędek (1999) pointed to the interesting observation that people suffering from depression perform some cognitive tasks well, while doing much worse in other, seemingly similar, tasks. For example, depressive subjects have problems with processing moderately structured materials, but none with low or highly structured materials (reviewed in von Hecker and Sędek 1999). Other studies have shown no increased sensitivity of depressed subjects to all negatively marked words, though they were observed to be more susceptible when socially threatening words were involved (Mathews et al. 1996, as cited in Taylor and John 2004). Inconsistent results are also provided by reports on the memory function of people with depression. Though many reports show the existence of general memory deficits in depressive subjects, others do not confirm any overall deterioration of memory, only confirming the existence of specific deficits, limited to specific tasks (for a review, see e.g., Ellwart et al. 2003). For example, Watkins and associates (Watkins et al. 2000) showed that depressive subjects remember negative words better than positive ones, but only in conceptually controlled tests, not in perceptually controlled ones.

One possible interpretation is that depression primarily affects effortful, elaborative processing, and not automatic, pre-attentive processes (cf. the “integrated theory” of Williams et al. 1997, as cited in Ellwart et al. 2003). This approach seems to be confirmed by recent reports on the memory function of depressive subjects. For example, in the studies of Ellwart and associates (2003; cf. also Taylor and John 2004), depressive subjects did not show deficits of implicit memory, but only of explicit memory, and only after cognitive interference between the phases of learning and recall. This was interpreted as evidence that the functioning of people with depression is not disturbed during the basic direct processing of information, and that problems arise only when an additional burden is placed on the cognitive system (cf. the theory of Kofta and Sędek 1998).

An interesting rationalization of cognitive deficits linked to depression was proposed by Sędek, Brzezicka, and von Hecker (described in Sędek et al. 2010). Their cognitive exhaustion model (e.g., Sędek et al. 1993, as reviewed in Sędek et al. 2010) explains why poorer function occurs during processing of complex information, and links cognitive function disturbances (in particular, those observed in depressive subjects) to failure to produce mental models (cf. Johnson-Laird 1983). Their theory assumes that people performing a complicated task try to extract important information from among unimportant stimuli, and to grasp the essence of the problem. In a controlled situation, they most often manage to create integrated mental representations of the problem, which helps them perform the task effectively. However, when there is no perceived control (e.g., in unsolvable problems), the efforts to create a general model are unproductive, and lead to no progress in task performance, while at the same time depleting the individual’s cognitive resources. According to the authors in question, this approach can be used successfully to explain cognitive deficits in depression. Due to ruminations and painful experience from the past, people with depression expect lack of control. They expect an unsolvable situation, leading to uncertainty. This, in turn, impairs their integration processes, their ability to produce mental models of complex problems, but without weakening their functioning in tasks requiring simple information processing or simple decisions (for a review, see Sędek et al. 2010). The expectations stemming from the cognitive exhaustion model have found empirical confirmation in studies involving depressive and non-depressive subjects (e.g., von Hecker and Sędek 1999; Sędek and von Hecker 2004).

The existence of cognitive changes in depression is well established. However, results are contradictory as concerns what happens during remission from depression. Older studies suggest that depression-related cognitive changes fade away during remission (e.g., Barnett and Gotlib 1988, as cited in Ilardi and Craighead 1999). More recent studies suggest that even people cured of depression manifest a special model of information processing (Atchley et al. 2007). For example, results showing that subjects after a 6-month remission from depression perform worse on tasks involving verbal memory and verbal fluency, compared to subjects who have never had depression (Neu et al. 2005; Biringer et al. 2005; as cited in Talarowska et al. 2009); nor was improvement observed in episodic memory (studies of Airaksinen and associates 2006, as cited in Talarowska et al. 2009). Williams et al. (2007) reviewed research on excessive generalization of memory in affective disorders, and reported that this feature of memory function persisted even in individuals who had experienced an episode of mood disturbance once, even if their mood at the time they were studied was not disturbed. Holmes and Pizzagalli (2007) reported on executive function deficits observed after depression symptoms had disappeared. Recently, it has even been suggested that cognitive susceptibility to depression may be genetically conditioned and could manifest itself in early childhood (see Hayden et al. 2008).

### Studies on the Comprehension of Notions by Subjects Suffering from Depression

Insofar as we are aware, the problem of conceptual process disturbances in depression is seldom undertaken in contemporary publications. Among recent studies it is hard to find projects that specifically study the comprehension of notions (e.g., temporal notions and names of emotions related to symptoms of depression) in depressive subjects. Though there are some studies comparing the perception of time by patients suffering from depression and healthy subjects (e.g., Mahlberg et al. 2008; Gil and Droit-Volet 2009; Sévigny et al. 2003), they are not concerned with *comprehension* of notions related to time, but only with time *perception*.

One promising method of studying the cognitive representations of selected notions in depressive subjects seems to be the analysis of the metaphorical conceptualization of these notions. It turns out that processing of metaphorical content is controlled by cognitive functions similar to those that are disturbed when the disease intensifies (especially working memory, cf. “Cognitive Disturbances in Depression Possibly Affecting the Comprehension of Notions”, “Linguistic and Psycholinguistic Premises for Studying Metaphorical Conceptualizations Produced by Depression Sufferers” sections.). This may indicate that there are differences in the metaphorical conceptualizations produced by healthy people and by patients suffering from depression.

Studies on how people suffering from various mental disorders process metaphorical content is currently a strongly developing trend in the psychological and psychiatric literature. Recent publications describe disturbances in the processing of metaphorical content in different mental and neurological diseases, including schizophrenia (e.g., Langdon et al. 2002, as cited in Rapp et al. 2007; Sponheim et al. 2003, cited in Argyris et al. 2007), Alzheimer’s disease (e.g., Papagno et al. 2003; cited in Argyris et al. 2007), Asperger syndrome (see Rapp et al. 2004), autism (e.g., Seitz 1996, as cited in Seitz 2005), and alexithymia (see Seitz 2005). To our knowledge, however, there is a lack of research on how people suffering from depression process metaphorical stimuli.

### Linguistic and Psycholinguistic Premises for Studying Metaphorical Conceptualizations Produced by Depression Sufferers

To study depression-related cognitive disturbances affecting the level of notions, we had to adopt an interdisciplinary theoretical framework, one that would include not only psychopathological theories, but also psycholinguistic and linguistic perspectives and methods. In its broad sense, the topic discussed here is linked with the relation between language and cognition, a problem that has been investigated for over half a century within cognitive psychology (Whorf 1956 vs. Fodor 1975; Kay and McDaniel 1978; Pinker 1994 for the cognition–language relation in the light of present-day opinions, see e.g., Vigliocco and Kleiner 2004; Rączaszek-Leonardi 2011; for the role of cognition in selected linguistic theories, see e.g., Butler 2008; for a review, see e.g., Bartczak 2009). In a narrower approach, the study of the metaphorical conceptualizations of notions requires dealing with the very vague and fuzzy definition of *metaphor* (for a review of theories and definitions of metaphorical language, see e.g., Bartczak 2009). When studying cognitive distortions at the notion level, observed in various mental disorders (whose etiology is often interpreted as the effect of disturbances in brain function), including depression, it seems appropriate to turn to neuropsychological theories of metaphor comprehension (e.g., Schnitzer and Pedreira 2005; see also Feldman 2006; Gibbs 2006). This approach is well represented by contemporary psycholinguistic theories on metaphorical language processing, in a trend to integrate linguistics with neuronal theories (Kravchenko 2006; cf. e.g., the neural theory of language, Feldman and Narayanan 2004; the network model of human language, Markošová 2008). Generally, neuropsychological theories of metaphor, which invoke neuropsychological arguments based on classical connectionist theories (Hebb 1949; Hayek 1952), assume that metaphorical thinking is primary. Put more simply, the main idea of neuropsychological theories of metaphor is that metaphorical thinking is conditioned by the architecture of the human brain (cf. e.g., Schnitzer and Pedreira 2005). Metaphors are described not as figures of speech, but as neuron maps connecting the network of the metaphorical vehicle with the network of the metaphor’s topic, e.g., the network of JOURNEY with the network of LOVE in the case of the well-known metaphor LOVE IS A JOURNEY. As metaphorical content is processed, an integrated circuit is immediately created in which both the vehicle and the topic are processed simultaneously (cf. Tendahl and Gibbs Jr. 2008). In the light of neuropsychological theories, the importance of metaphor for human cognition is explained with reference to the specificity of learning processes. Connectionist theories describe them as the creation, strengthening, or modification of synaptic connections on the basis of repetitive stimulation (the weakening of relations takes place in a similar way). Knowledge is gathered most rapidly and efficiently if the absorption of information requires minimal, and not substantial, changes in the network of connections (Goldbaum 2001, as cited in Schnitzer and Pedreira 2005). Thus, by enabling more complicated experiences to be expressed in terms of simpler and more basic ones, metaphor becomes the fundamental tool of cognition. In recent years there has been a marked increase in research on metaphor comprehension using neuroimaging methods (one of the earliest is that of Bottini et al. 1994; for studies on the processing of metaphorical word pairs, see e.g., Lee and Dapretto 2006; Mashal et al. 2007; for research on metaphorical sentences, see e.g., Eviatar and Just 2006; Rapp et al. 2007; Argyris et al. 2007). The results most often show that metaphors are processed through a special neuronal mechanism (for studies with the use of fMRI, see e.g., Shibata et al. 2007). There is also evidence that different kinds of metaphorical mappings reflect different specific brain networks (cf. e.g., the basic metaphor theory; Seitz 2005).

Regardless of which definition of metaphor is chosen, studying the metaphorical conceptualization of notions in people suffering from depression, and in healthy subjects, necessitates the assumption of inter-individual variability in metaphorical processing. Although recent years have seen many cases proving that individual differences could serve as performance predictors for many language tasks (for a review, see Blasko 1999), the hypothesis that selected qualities of the subject affect the processing of metaphorical content has hitherto seen little empirical verification (cf. the discussion in Blasko 1999). Much greater interest has been shown in the influence of selected qualities of metaphors themselves (e.g., familiarity, accuracy, etc.) on the process of their comprehension (for an overview, see e.g., Bartczak 2009). It is still not clear what particular variables from the group of individual differences would cause differences in metaphorical content processing. It seems unlikely that these are cultural or inter-linguistic differences (the results of neuropsychological studies involving subjects speaking English, German, Mandarin Chinese, and Japanese have been reported by Shibata et al. 2007). The more probable track could be individual differences in working memory capacity and executive function capabilities.

One example of a theoretical model predicting how working memory affects metaphor processing is Kintsch’s predication model (Kintsch 2000, 2001). This model presents the understanding of metaphorical expressions as a process of spreading activation in a self-inhibiting semantic network consisting of the predicate $P$, argument $A$ (or the vehicle and topic of the metaphor), and their $n$ closest neighbors. Kintsch’s model assumes that individual differences in working memory capacity, and in executive function (particularly inhibition processes), will affect the processing of metaphorical utterances. Someone with working memory deficits (a) may not have sufficient resources to activate an adequately extensive semantic network and (b) will do worse at inhibiting distinctive but irrelevant qualities of the predicate, which usually leads such a person to provide an interpretation of metaphorical content more slowly, and this interpretation tends to be of poorer quality (cf. also Blasko 1999; Gernsbacher et al. 2001). An example of a different approach to explain differences in performance of tasks requiring metaphor processing, in terms of working memory, is the capacity theory of language (Just and Carpenter 1992). In this theory, working memory is thought to affect both the speed and effectiveness of metaphorical content comprehension. The role of working memory in metaphor processing can also be approached through Glucksberg’s class-inclusion model (Glucksberg and Keysar 1990; Glucksberg 2001, 2003). This model assumes that metaphors are understood as category statements. For example, the metaphor *Cigarettes are time bombs* categorizes the topic of the metaphor (*cigarettes*) as belonging to the category—formed ad hoc—of “objects that are deadly over time,” a typical representative being the metaphor’s vehicle (*a time bomb*). This superordinate ad hoc category is activated in the metaphor comprehension process, enabling the subject to provide the correct interpretation of the metaphorical meaning. Note that the class-inclusion model, like Kintsch’s model, emphasizes the important role of working memory mechanisms in the correct interpretation of metaphorical statements. Creating an *ad hoc *category appropriate to the given context requires highlighting those qualities of the vehicle that are key to the metaphor’s meaning (e.g., *being dangerous to health and life*) and inhibiting those that fit in with the basic category, but are irrelevant for the meaning of the metaphor (e.g., *a terrorist tool*). The role of these mechanisms (also known as enhancement and suppression effects), especially the inhibition mechanism, has been confirmed by studies such as those of Gernsbacher et al. (2001).

The prediction stemming from the foregoing theoretical models, ascribing major importance to working memory in metaphor processing, has been confirmed empirically by Chiappe and Chiappe (2007) on the comprehension and production of metaphors by healthy adults. Working memory was found to affect the processing of metaphorical content, irrespective of a person’s vocabulary and reading level. The influence of working memory on the assessment and interpretation of metaphors has also been confirmed by Blasko and Trich (1997, cited in Blasko 1999). The subjects in their study were given a pretest (the reading span task, Daneman and Carpenter 1980, as cited in Blasko 1999) and divided into low, medium, and high working memory span groups. They were then asked to read some stimulus metaphors and to interpret them in their own words. An evaluation of the subject’s interpretation of these metaphors, performed by competent raters using a 7-point scale, showed that the best (most exhaustive and in-depth) interpretations were provided by subjects with the greatest working memory span. Similar conclusions were drawn in a recent study on metaphorical language comprehension in subjects with Parkinson’s disease (Monetta and Pell 2007). This study, which used the metaphor comprehension task (Gernsbacher et al. 2001), showed that only subjects with working memory deficits did worse in metaphor processing tasks.

Taking into account the results of empirical studies outlined above, and the fact that memory deficits are present in patients with depression, one can expect differences between healthy and depressive subjects in performance of tasks involving metaphorical stimuli. However, according to our knowledge, this problem has not yet been considered in the contemporary literature yet.

## A Study on the Metaphorical Conceptualizations of PAST, FUTURE, JOY, SADNESS, and HAPPINESS in Depressive and Non-depressive Subjects

### Research Questions and Hypotheses

Considering (a) the lack of research on the notion level of cognitive distortions in people suffering from depression, (b) unresolved discussions on how cognitive changes persist during remission from depression, (c) the similarity between the cognitive functions responsible for metaphorical language processing and those that are disturbed in depressive subjects, (d) the need to investigate aspects as not yet included in research on metaphor (inter-individual differences in processing of metaphorical stimuli, with a special focus on the low mood variable; the metaphor valence dimension), and (e) the success of previous research on metaphor processing by patients suffering from different mental disorders, the following questions are posed:

1. Is depression correlated with changes in the cognitive representation of notions (as indicated by the valence and number of metaphorical conceptualizations)?
2. Do depressive changes in the cognitive representation of notions recede during remission from the disease?

Based on:

- theoretical premises (including the cognitive theory of depression, Beck 1963, 1967, especially the content-specificity hypothesis, Beck 1976; the neuropsychological theory of metaphor, e.g., Schnitzer and Pedreira 2005; the predication model, Kintsch 2000, 2001);
- the results of research on metaphor processing, suggesting that proper comprehension and production of metaphorical expressions requires an efficient working memory mechanism (e.g., Chiappe and Chiappe 2007; Monetta and Pell 2007);
- the results of research on the cognitive function of depressive subjects, suggesting working memory impairment and changed attention function during depression (e.g., Fossati et al. 1999; von Hecker and Sędek 1999; von Hecker and Meiser 2005);
- results suggesting that even subjects cured of depression manifest a specific information processing pattern (e.g., Atchley et al. 2007; cf. also the overview in Holmes and Pizzagalli 2007; Talarowska et al. 2009);

the following hypotheses are proposed:

1. Depression is correlated with changes in the cognitive representation of notions, in particular:
  - Depressive subjects produce fewer metaphors of a given notion than healthy people;
  - Depressive subjects produce relatively more metaphors of notions with a negative valence than healthy people;
  - Compared to the cognitive representations of notions produced by healthy people, the cognitive representations of neutral and positive notions produced by depressive subjects will have a more negative valence, and the cognitive representations of negative notions—a more positive valence;
2. A depressive pattern of cognitive representation is also observable during remission of the disorder (cf. e.g., Atchley et al. 2007; and the review in Holmes and Pizzagalli 2007).

### Selection of Notions for Analysis

The choice of notions for this study was based on the theoretical characterization of depressive disorders. Two temporal notions (PAST and FUTURE) as well as three names of emotions (JOY, SADNESS, and HAPPINESS) were selected. The reason is that many sources listing the symptoms of depression use these terms to describe cognitive distortions and affective disturbances in people with depression. It is believed that depressive people perceive their past mainly as a source of failure and depict their future in dark colors, while the dominating mental state of depressive people is sadness, and they are unable to feel joy or to be happy (e.g., Pużyński 2002, p. 360; Rosenhan et al. 2003, p. 272). Because PAST, FUTURE, JOY, SADNESS, and HAPPINESS are notions used to describe cognitive and affective disturbances linked to depression, it may be suspected that their cognitive representations could be different in healthy and depressed subjects.

### Methods

#### Participants

This study was conducted from June to December 2010 and involved three groups of adults. The first (experimental group, E) comprised 30 subjects suffering from depression (24 women, 6 men; $M_{age} = 44.3$, range 21–77)—patients of psychiatric wards and outpatient clinics in Warsaw hospitals (Institute of Psychiatry and Neurology and Wolski Hospital) with a diagnosis of F32.1 and F33.1 according to ICD-10 criteria (Pużyński and Wciórka 1997).^1^ The second (control group, C) comprised 30 subjects who had never suffered from depression (24 women, 6 men; $M_{age} = 44.6$, range 23–83)—medical and non-medical staff of the institutions concerned. The third (remission group, R) were patients from outpatient clinics who had had a depressive episode and were in remission (with a diagnosis of F33.4 or F32 in their medical history based on ICD-10^2^). As far as we know, they received similar kinds of treatment: They had just stopped, or were in the course of gradual discontinuation of, antidepressants. The third group was the smallest and included 12 subjects (9 women, 3 men; $M_{age} = 49.1$, range 23–78). In the study’s 6 months it proved impossible to reach a larger number of people meeting the criteria for group R (no symptoms of depression at the time of the study, a depressive episode in the past). The demographics for the three participant groups are presented in Table 1.

**Table 1:** Demographics of the three participant groups

	Group E	Group C	Group R
$n$	30	30	12
Age	$M = 44.3$	$M = 44.6$	$M = 49.1$
	(range 21–77, $\text {SD}=14.22$)	(range 23–83, $\text {SD}=17.88$)	(range 23–78, $\text {SD}=17.82$)
*Sex*			
Women	24	24	9
Men	6	6	3
*Education*			
Higher education	13	6	7
Baccalaureate	17	24	5
*Place of residence*			
Country	3	2	2
Town:$^{a}$ up to 20,000	2	2	0
20,000–49,999	1	0	0
50,000–99,999	1	2	0
100,000–499,999	2	2	0
500,000 and more	21	22	10

$\text {E}=\text {experimental group}$ (subjects suffering from depression); $\text {C}=\text {control group}$ (subjects who have never suffered from depression); $\text {R}=\text {remission}\text {group}$ (patients who had a depressive episode in the past but are currently in remission).

$$^{\mathrm{a}}$$ Population

The selection of subjects for groups E and R was made in cooperation with four psychiatrists, who singled out patients with the required diagnosis and asked for their consent to take part in a study on notions. The criterion for assigning the subjects to a given group was the medical diagnosis based on an in-depth interview and taking into account their result in the Beck Depression Inventory (BDI; Beck 1973; Beck and Beamesderfer 1974; Beck et al. 1987; cf. also Parnowski and Jenajczyk 1977). As with other studies involving depressive subjects, it was agreed that an individual suffered from depression if their result was equal to or higher than 10 (Beck et al. 1987; Ruscio and Ruscio 2002; see also Fajkowska and Marszał-Wiśniewska 2006). The average BDI value in group E was 25.95 (range 18–48), strongly distinguishing it from the other two groups: In groups C and R the average BDI values were similar, $\text {BDI}=5.0$ (range 0–9) and $\text {BDI}=5.25$ (range 1–9), respectively.

#### Materials

Among other things, the subjects filled in the Questionnaire of the Metaphorical Conceptualization of a Notion (QMCN; cf. Bartczak 2009b)^3^. The QMCN comprised 60 sentences related to the *past*, *future*, *joy*, *sadness,* and *happiness* (12 per notion). The subject’s task was to “read the sentences and then assess how accurately they describe the notions of *past*, *future*, *joy*, *sadness,* and *happiness*.” The subjects made their assessment on a 5-point relevance scale (from 1—*very inaccurate sentence*, to 5—*very accurate sentence*).

#### Metaphoricity, Valence, and Conventionalization Ratings

The QMCN is a tool with a solid empirical foundation. The sentences were taken from narratives written by depressive (EP; $n = 10$) and non-depressive (CP; $n = 10$) adults during a pilot study conducted in 2007 and 2008. The subjects were asked to write short stories on the topics: *The Past*, *The Future*, *Joy*, *Sadness*, and *Happiness* (for detailed information on the tools, procedure, and results, see Bartczak 2008). Sentences about the respective notions were taken from these stories. The selected sentences were then given to competent raters for assessment. The valence and conventionalization of the sentences was judged by 25 students from the University of Warsaw’s Institute of Applied Linguistics (20 women and 5 men aged 19–22, $M_{age} = 19.2$). They were asked to read each sentence and to decide if its meaning is positive, neutral, or negative (assessment of valence), and to assess how often this type of sentence appears in everyday speech (assessment of conventionalization). The judges marked their replies on two 5-point scales: the valence scale (from 1—*strongly negative sentence*, to 5—*strongly positive sentence*) and the conventionalization scale (from 1—*sentence very seldom found in everyday speech*, to 5—*sentence very often found in everyday speech*). Sixty items were chosen for the QMCN (12 for each notion), including 34 statements by depressive patients and 26 by healthy people. The sentences included in the QMCN were those with the lowest and highest marks on the valence and conventionalization scales (for examples, see “Appendix”, Table 2). When the judges’ average marks were the same for two or more items, the lower standard deviation was taken into account. In the next stage, the 60 items selected for the QMCN were assessed for their metaphoricity by a different group of competent raters. These were 40 students from a psycholinguistics course at the University of Warsaw’s Faculty of Psychology, 33 women and 7 men ($M_{age} = 20.92$, *range* 19–26). The judges were asked to read the sentences and then to “decide if they are metaphorical or not.” The instruction told them to treat as metaphorical those statements in which the meaning is more concrete than the original notion (examples from the instruction: very metaphorical, concrete usages: *The past is a little girl in a blue dress*; *Happiness tastes like hot chocolate*; very non-metaphorical, abstract usages: *The past is an unknown*; *Sadness is a negative emotion*). The judges marked their replies on a 5-point scale (from 1—*very non-metaphorical, abstract meaning*, to 5—*very metaphorical, concrete meaning*). Examples of QMCN items with their marks on the metaphor scale are shown in Appendix Table 3.

The sentences were placed in the questionnaire in random order. The QMCN’s reliability is high: Cronbach’s alpha for the whole questionnaire is .787, and for the individual subscales: .720 (metaphoricity), .796 (valence), and .730 (conventionalization).

**Table 2:** Sample items from the questionnaire of the metaphorical conceptualization of a notion: assessment of valence and conventionalization

Valence	
Negative	Dla mnie przeszłość była ciężka i przykra. [For me the past was tough and painful.] ($M= 1.2$; $\text {SD}=0.45$)
	Przyszłość to utrata czucia w nogach, ślepota i uśnięcie. [The future is losing sensation in your legs, blindness, and going to sleep.] ($M = 1.2$; $\text {SD}=0.45$)
	Czasem uczucie smutku jest przytłaczające. [Sometimes the feeling of sadness is overwhelming.] ($M = 1.2$; $\text {SD}=0.45$)
Positive	Radość to słońce i kwiaty. [Joy is sunshine and flowers.] ($M = 4.8$; $\text {SD}=0.45$)
	Przyszłość może nieść w sobie bardzo, bardzo dobre niespodzianki. [The future could bring very, very nice surprises.] ($M = 4.8$; $\text {SD}=0.45$)
	Smuteczek pozwala pobyć chwilę w ciszy, posłuchać jak dżdżownica ryje ziemię. Pozwala skupić się na małostkach: na motylach, na ludziach, którzy potrzebują pomocy${\ldots }$ [Sadness enables you to sit in silence for a while, listen to an earthworm digging through the dirt. It allows you to focus on little things: butterflies, people who need help${\ldots }$] ($M = 4.2$; $\text {SD}=0.45$)

Conventionalization	
Low	Smutek jest takim małym stworzonkiem, które można zamknąć w dłoniach i przyjemnie łaskocze—ma puchate futerko. [Sadness is a little creature you can hold closed in your hands and it tickles pleasantly—it has fluffy fur.] ($M = 1.2$; $\text {SD}=0.45$)
	Szczęście to tęczowe bańki powietrza. [Happiness is rainbow-colored bubbles of air.] ($M = 1.4$; $\text {SD}=0.55$)
	Przyszłość jest mniej kanciasta i narzucająca się. [The future is less angular and overbearing.] ($M = 1.4$; $\text {SD}=0.55$)
High	Radość to być z kimś, kogo się kocha. [Joy is being with someone you love.] ($M = 5$; $\text {SD} = 0$)
	Przyszłość będzie taka, jaką ją zbudujemy sami. [The future will be how we build it ourselves.] ($M = 4.8$; $\text {SD}=0.45$)
	Szczęście to rodzina, żona, dzieci. [Happiness is family, wife, children.] ($M = 4.6$; $\text {SD}=0.55$)

The values in brackets represent the judges’ average ratings and the standard deviation

**Table 3:** Sample items from the questionnaire of the metaphorical conceptualization of a notion: assessment of metaphoricality

	Items rated as being the least metaphorical	Items rated as being the most metaphorical
PAST	Przeszłość to coś, co już minęło. [The past is something that has already gone by.] ($M = 1.82$; $\text {SD}=0.90$)	Przeszłość jest otwartą księgą pełną strachu i nieprzyjemnych wspomnień. [The past is an open book full of fear and unpleasant memories.] ($M = 4.25$; $\text {SD}=1.05$)
	Przeszłość to czas, który minął. [The past is time that has gone by.] ($M = 1.90$; $\text {SD}=1.15$)	Gdyby tak można było zamknąć przeszłość raz na zawsze i kłódce pozwolić zardzewieć${\ldots }$ [If only you could only lock the past away once and for all and let the padlock rust${\ldots }$] ($M = 4.42$; $\text {SD}=0.88$)
FUTURE	Przyszłość to to, co jeszcze przed nami. [The future is whatever is still ahead of us.] ($M = 1.85$; $\text {SD}=0.89$).	Przyszłość jest mniej kanciasta i narzucająca się. [The future is less angular and overbearing.] ($M = 4.0$; $\text {SD}=1.26$)
	Przyszłość jest pełna optymizmu. [The future is filled with optimism.] ($M = 2.12$; $\text {SD}=0.94$)	I tak przyszłość to czarna dziura. [And so the future is a black hole.] ($M = 4.22$; $\text {SD}=1.07$)
JOY	Radość zawsze może się skończyć. [Joy can always end.] ($M = 2.08$; $\text {SD}=1.03$)	Radość rozpiera dawców radości, jakby miała się wylać z nich. [Joy makes its givers burst with joy, as if it were going to pour from them.] ($M = 4.2$; $\text {SD}=0.91$)
	Mnóstwo rzeczy sprawia mi radość, czyli wywołuje uśmiech na twarzy. [Lots of things give me joy, meaning they bring a smile to my face.] ($M = 2.15$; $\text {SD}=1.33$)	Czasem radość bywa skrywana pod grubszą warstwą skóry, ochronnego płaszcza—pancerza. [Sometimes joy is hidden under a thicker layer of skin, a protective coat—armor.] ($M = 4.38$; $\text {SD}=0.92$)
SADNESS	Smutek dotyka nas, jak stracimy bliską osobę. [Sadness touches us when we lose someone dear.] ($M = 2.12$; $\text {SD}=1.24$)	Mam wrażenie, że gdyby malarz chciał spersonalizować smutek, powinien mnie poprosić o pozowanie. [I have a feeling that if a painter wanted to personalize sadness, he should ask me to pose for him.] ($M = 3.98$; $\text {SD}=1.02$)
	Czasem uczucie smutku jest przytłaczające. [Sometimes the feeling of sadness is overwhelming.] ($M = 2.22$; $\text {SD}=1.07$)	Smutek jest takim małym stworzonkiem, które można zamknąć w dłoniach i przyjemnie łaskocze—ma puchate futerko. [Sadness is a little creature you can hold closed in your hands and it tickles pleasantly—it has fluffy fur.] ($M = 4.6$; $\text {SD}=1.08$)
HAPPINESS	Szczęście zazwyczaj krótko trwa, albo wcale się nie zdarza. [Happiness usually lasts a short time, or it doesn’t happen at all.] ($M = 1.8$; $\text {SD}=0.99$)	Szczęście to właśnie te ulotne bańki powietrza, które pękają niespodziewanie i szybko mijają. [Happiness is those fleeting bubbles of air that burst unexpectedly and are quickly gone.] ($M = 4.45$; $\text {SD}=1.08$)
	Szczęście odczuwamy zawsze wtedy, kiedy uda nam się pokonać trudności. [We feel happiness whenever we manage to overcome difficulties.] ($M = 2.0$; $\text {SD}=1.09$)	Szczęście to tęczowe bańki powietrza. [Happiness is rainbow-colored bubbles of air.] ($M = 4.52$; $\text {SD}=0.93$)

The values in brackets represent the judges’ average ratings and the standard deviation

#### Procedure

All procedures were carried out according to the Declaration of Helsinki and were approved by the ethical committee of the University of Warsaw’s Faculty of Psychology. Participants were tested individually. The subjects were asked for their consent to take part in a research project on the comprehension of notions. Upon giving their consent, they received forms with the tools and instructions, together with a token gift in the form of a pen with the University of Warsaw logo. Due to depressive patients’ greater tendency to tire easily, the instructions offered the possibility of taking a break if the subject felt tired, and then to continue at a later time. The subjects filled in the questionnaire at home and brought it with them on their next visit. It took them about 45 min to fill in the questionnaire. The managers of the units where the study was conducted agreed to their patients’ and employees’ participation in the project. Depressive patients were chosen for the study in collaboration with the psychiatrists treating them; participation in the project did not interfere with the patients’ therapy.

#### Data Analysis

Independent-measures $t$ tests were used to examine the existence of significant inter-group differences. The normality of the distributions was checked using the Kolmogorov–Smirnov test. Each dimension (metaphoricity, valence, conventionality) was analyzed separately.

Regarding the metaphoricity dimension, from among the 60 sentences in the tool, the items which the judges had given marks well over the average on the metaphoricity scale (2.9 or more on the 5-point scale) were recognized as metaphorical. This criterion was fulfilled by a total of 25 items, five each for PAST and FUTURE, three for JOY, eight for SADNESS and four for HAPPINESS.

Similarly, positive valence was given to those sentences that the judges had given clearly higher than average marks on the valence scale (2.9 and more). This condition was fulfilled by 31 of the 60 items on the QMCN, including five for PAST, six for FUTURE, seven for JOY, four for SADNESS, and nine for HAPPINESS.

Finally, regarding the conventionality dimension, the sentences recognized as conventional were those which the competent judges had assessed clearly above average on the conventionalization scale (2.9 or more on the 5-point scale), similarly to the choice of items with high metaphoricity and high valence. This criterion was met by just over half of all the items on the QMCN (36 out of 60), including seven sentences each for PAST and JOY, nine each for FUTURE and HAPPINESS, and four for SADNESS.

We calculated the QMCN scores as follows: In the case of PAST and the metaphoricity dimension, five QMCN sentences had been found highly metaphorical. The participant rated each sentence on the 5-point accuracy scale (i.e., each of the five sentences could be rated at least 1 and maximally 5). All the ratings given were added up, so that regarding the PAST and the metaphoricity dimension, it was possible to receive ratings from 5 to 25. Similarly, in the case of SADNESS and the conventionality dimension, the ratings could develop from 4 to 20 (because four sentences for SADNESS had been found highly conventional). The scores were calculated in the same way for all three dimensions and all of the notions.

## Results

### Results for Metaphoricity: Checking Hypotheses 1a and 1b

Hypotheses 1a and 1b stated that (a) depressive subjects would produce fewer metaphors of a given notion than healthy people and that (b) depressive subjects would produce relatively more metaphors of notions with a negative valence than healthy people. Taking into account the features of the QMCN, the metaphor production variable in our study is operationalized as the rating of metaphorical sentence accuracy.

Contrary to the expectations of Hypothesis 1a (which predicted problems with processing of metaphorical content by depressive subjects), metaphoricity was not an aspect that strongly differentiated the replies of the groups in the study. Taking into account all the items of the QMCN, without splitting them into sentences related to the individual notions, all the groups more often rated as accurate statements with an average level of metaphoricity (the average intensity of the “metaphoricity” quality for the 20 items chosen as being the most accurate by groups E, C, and R was 2.74, 2.42, and 2.38, respectively), and as inaccurate—those with a higher than average metaphoricity (the average intensity of the quality of “metaphoricity” for the 20 items chosen as being the least accurate: E: 3.29, C: 3.47, and R: 3.25).

Let us look at the results when the items of the QMCN are split into statements on the individual notions. An analysis with the $t$ test confirmed the initial observations: Depression sufferers did not choose metaphorical items as being accurate significantly less often than subjects from the control group (no confirmation of Hypothesis 1a). Furthermore, in the case of three notions—PAST, FUTURE, and SADNESS—it turned out that depressive subjects significantly more often than healthy subjects chose sentences with a high level of metaphoricity as being accurate. The result for SADNESS, E: $M = 24.90$, $\text {SD}=4.71$; C: $M = 18.37$, $\text {SD}=5.20$, $t(58) = 5.10$, $p< .001$, confirms Hypothesis 1b and its prediction of intensified metaphorical processing of negatively marked notions by depressive subjects. Higher marks for metaphorical sentences regarding PAST and FUTURE given by the experimental group; PAST—E: $M = 15.83$, $\text {SD}=2.70$; C: $M = 11.83$, $\text {SD}=2.84$, $t(58) = 5.58$, $p < .001$; FUTURE—E: $M = 16.10$, $\text {SD}=1.95$; C: $M = 14.13$, $\text {SD}=2.36$, $t(58) = 3.52$, $p < .001$; can also be considered as agreeing with the hypothesis, if we assume that these notions have a negative valence for patients suffering from depression.

**Fig. 1:**
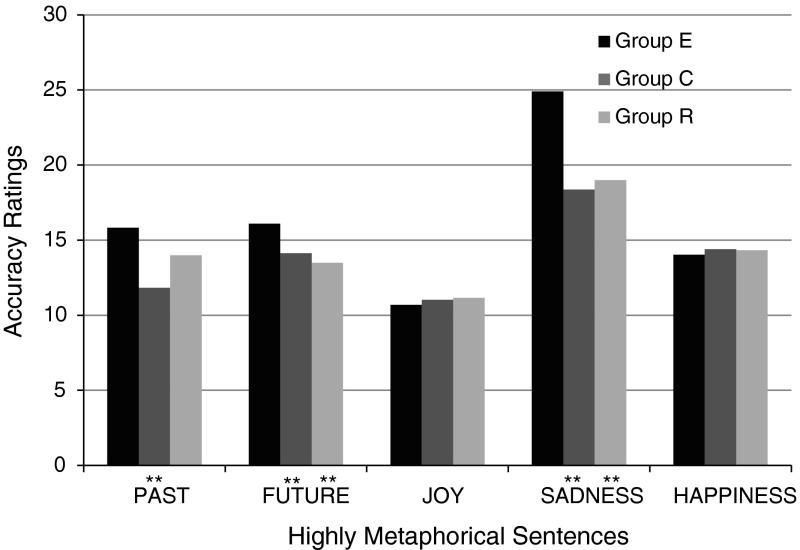
Summed averages of metaphorical item ratings in the group of depressive subjects (E), healthy subjects (C) and subjects in remission (R)

In summary, the pattern of results obtained in the QMCN does not confirm Hypothesis 1a and its prediction of patients with depression having problems with processing metaphorical content. On the contrary, taking into account the items for all the notions, depressive subjects chose metaphorical sentences as being accurate significantly more often ($M = 81.57$, $\text {SD}=7.92$) than subjects from the control group, $M = 69.77$, $\text {SD}=9.98$, $t(58) = 5.07$, $p < .001$. At the same time, the QMCN results provide evidence in support of Hypothesis 1b regarding intensified metaphorical processing of negatively marked notions. The differences between groups on the metaphoricity scale are shown in Fig. 1.

### Results for Valence: Testing Hypothesis 1c

**Fig. 2:**
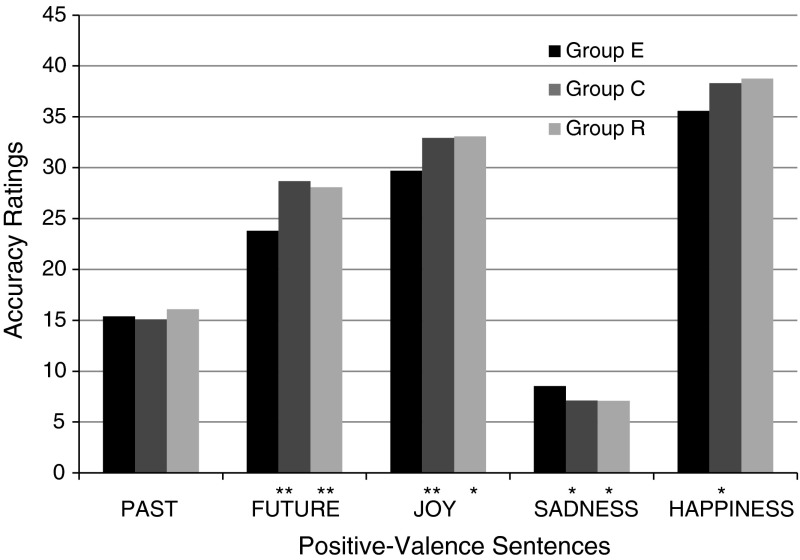
Summed averages of high-valence item ratings in the group of depressive subjects (E), healthy subjects (C) and subjects in remission (R)

A comparison of the results for the depressive group and the control group brought strong confirmation of Hypothesis 1c regarding the difference in the valence of the cognitive representations produced by depressive and healthy subjects (see Fig. 2). Compared to the control group, subjects from the experimental group less often chose as accurate the items related to a neutral notion: FUTURE, E: $M = 23.80$, $\text {SD}=4.04$; vs. C: $M = 28.67$, $\text {SD}=3.15$, $t(58) = -5.20$, $p < .001$; and to positive notions: JOY, E: $M = 29.70$, $\text {SD}=5.27$; vs. C: $M = 32.93$, $\text {SD}=2.75$, $t(58) = -2.98$, $p = .004$; and HAPPINESS, E: $M = 35.57$, $\text {SD}=6.86$; vs. C: $M = 38.30$, $\text {SD}=4.99$, $t(58) = -1.76$, $p = .04$, which had a high valence. In the case of the negatively marked notion (SADNESS), as expected, the correlation was the opposite. Patients with depression gave significantly higher marks to sentences with high valence ($M = 8.53$, $\text {SD}=2.57$) than subjects from the control group, $M = 7.10$, $\text {SD}=2.31$, $t(58) = 2.27$, $p = .027$. The PAST was the only notion for which inter-group differences did not reach a statistically significant level.

### Results for Conventionalization

Apart from the metaphoricity and valence of statements, the QMCN controlled one more dimension: conventionalization. Many contemporary studies on metaphorical language processing have provided evidence that conventionalization, the frequency with which a given structure occurs in a language, is one of the factors most strongly affecting metaphor processing (cf. e.g., Blasko and Connine 1993; Giora et al. 2000; Keysar et al. 2000). Considering the cognitive distortions occurring in people suffering from depression, and premises suggesting a difference in the valence of representations produced by depressive and healthy subjects, one could conclude that depressive subjects would be more likely than subjects from the control group to choose as accurate those items that have a lower level of conventionalization, and are used less often in speech.

**Fig. 3:**
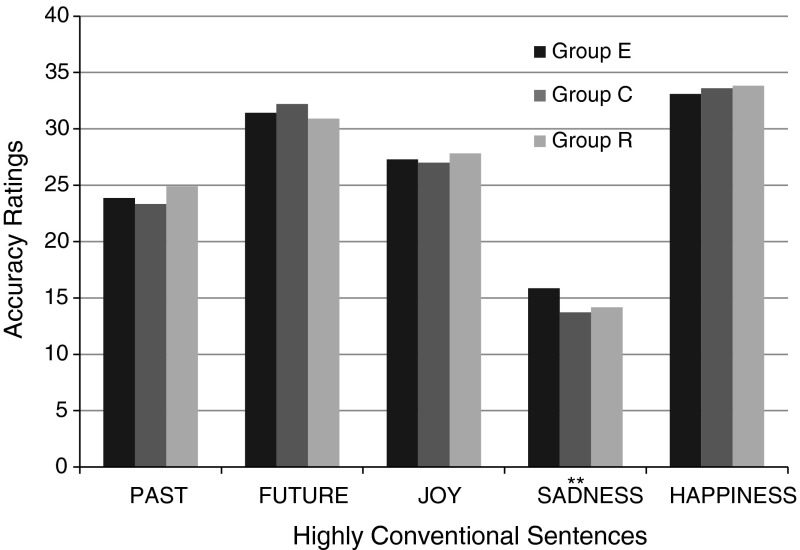
Summed averages of high-conventionalization item ratings in the group of depressive subjects (E), healthy subjects (C) and subjects in remission (R)

Contrary to expectations, conventionalization did not turn out to be an aspect strongly differentiating the replies of the groups in the study. Regardless of the group they were from, subjects more often chose items with a high rather than low conventionalization as being accurate (the average intensity of the quality of “conventionalization” for the 20 items chosen as being the most accurate by groups E, C, and R was: 3.71, 3.97, and 3.89, respectively). The difference in assessment of conventional sentences on SADNESS between groups E and C was the only significant inter-group difference (cf. Fig. 3). Subjects suffering from depression assessed these items as being more accurate ($M = 15.87$, $\text {SD}=2.39$) than subjects from the control group, $M = 13.73$, $\text {SD}=1.84$, $t(58) = 3.88$, $p < .001$. This result contradicts the expectation that people with depression would choose less conventional items than healthy people.

### Comparing Results of Depressive Subjects and Participants During Remission from Depression: Checking Hypothesis 2

Analysis of the QMCN results revealed significant differences between the replies of the experimental and control groups, in terms of both valence and metaphoricity.

Hypothesis 2 assumed that similar differences would be observed between the results of healthy subjects and those in remission. However, the results ran contrary to expectations. The assessments of group R differed significantly from those of group E and—in addition—they were very similar to the assessments of group C. Strikingly, in the case of four out of the five notions in the study (PAST, FUTURE, SADNESS, HAPPINESS) it even turned out that four items chosen as the most accurate were the same in both groups. An analysis of the replies of all three groups showed that all the observed statistically significant differences between the results of depressive subjects and those in remission are also observed between the results of depressive and healthy subjects. This is all the more interesting in that statistically significant differences between groups E and R concerned exactly the same aspects and the same notions as the differences between groups E and C. In other words, subjects during remission from depression replied almost exactly the same as subjects from the control group, but at the same time significantly differently from patients suffering from depression (results for metaphoricity—cf. Fig. 1, results for valence—cf. Fig. 2, results for conventionalizatio—cf. Fig. 3).

Like healthy subjects, members of group R assessed metaphorical sentences as less accurate ($M = 72.00$, $\text {SD}=6.07$) than depressive subjects, $M = 81.57$, $\text {SD}=7.92$, $t(40) = 3.76$, $p < .001$. Taking into account individual notions, subjects during remission from depression, like those from the control group, gave lower marks than depressive subjects to metaphorical sentences about the FUTURE, E: $M = 16.10$, $\text {SD}=1.95$; vs. R: $M = 13.50$, $\text {SD}=1.62$, $t(40) = 4.07$, $p < .001$; and SADNESS, E: $M = 24.90$, $\text {SD}=4.71$; vs. R: $M = 19.00$, $\text {SD}=3.19$, $t(40) = 3.98$, $p < .001$.

For valence, again the replies of group R were similar to those of group C. Subjects in remission gave higher marks to positive sentences about FUTURE and JOY than depressive patients, FUTURE—E: $M = 23.80$, $\text {SD}=4.04$; vs. R: $M = 28.08$, $\text {SD}=2.02$, $t(40) = -3.49$, $p < .001$; JOY—E: $M = 29.70$, $\text {SD}=5.27$; vs. R: $M = 33.08$, $\text {SD}=2.19$, $t(40) = -2.14$, $p = .019$ (one-tailed). In the case of SADNESS, similarly to the comparison between the results of groups E and C, an opposite correlation was observed: Members of group R gave lower marks to positive sentences about SADNESS ($M = 7.08$, $\text {SD}=1.88$) than subjects from group E, $M = 8.53$, $\text {SD}=2.57$, $t(40) = 1.77$, $p = .04$ (one-tailed).

Analysis of the results for the third dimension—conventionalization—also showed a similarity between the replies of groups C and R. Like members of group C, subjects during remission from depression gave lower marks to conventional sentences about SADNESS ($M = 14.17$, $\text {SD}=1.19$) than depressive patients, $M = 15.87$, $\text {SD}=2.39$, $t(40) = 2.34$, $p = .024$.

The following differences between groups E and R did not reach the level of statistical significance (though these differences were significant in the case of the results for groups E and C): Differences in assessment of metaphorical sentences about PAST (group R even gave these items significantly higher marks than group C, cf. E: $M = 11.83$, $\text {SD}=2.84$; vs. R: $M = 14.00$, $\text {SD}=2.58$, $t(40) = -2.28$, $p = .028$) and differences in assessment of positive sentences about HAPPINESS.

## Discussion

### Conclusions

#### Testing Hypothesis 1a

The overall results definitely did not confirm Hypothesis 1a and its prediction that people suffering from depression would have problems with processing metaphorical content: All groups more often chose as accurate those statements that had an average level of metaphoricity. This result can be considered surprising, in as much as earlier research on mechanisms of producing and understanding metaphors (e.g., Chiappe and Chiappe 2007; Monetta and Pell 2007) clearly suggested that working memory capacity was a major factor responsible for effective processing of metaphorical content, while research on the cognitive function of people suffering from depression provided evidence of disturbances of that memory in depressive subjects (e.g., Fossati et al. 1999; von Hecker and Meiser 2005). On the other hand, this lack of confirmation of Hypothesis 1a provides some interesting theoretical conclusions, both for models of metaphorical processing and for concepts explaining cognitive difficulties observed in patients with depression.

Taking into account theories related to metaphor processing, the result obtained could be considered as support for theories that promote the automatic, unconscious, and effortless nature of metaphorical content processing. If we assume that people with depression perform worse in tasks requiring complex processing of information, while at the same time tests requiring metaphorical processing (e.g., the QMCN) show no differences in performance between the depressive and control groups, there is reason to assume that processing of metaphorical content takes place automatically and without effort. Therefore, the results obtained here contradict the psychological plausibility of the standard pragmatic model (Grice 1975, reviewed in Blasko 1999), which assumes that metaphor processing requires more effort than processing of literal statements, because the appropriate non-literal interpretation takes place only after the contextually inadequate literal interpretation has been discarded. Thus, the results are compatible with many results of contemporary studies suggesting that the process of creating and understanding metaphors occurs just as automatically and quickly as with literal statements (e.g., Gernsbacher et al. 2001; Glucksberg 2003). The conclusions from the present study are also in agreement with the implications of neuropsychological theories of metaphor (e.g., Schnitzer and Pedreira 2005) which assume that metaphorical thinking is natural for human beings and is the result of the way the brain is organized and functions.

Leaving aside models of metaphor comprehension, the result of the present study is also interesting from the point of view of theories explaining cognitive deficits in depression. It seems possible to treat it as an argument in favor of models predicting that depression-related cognitive deficits will only become apparent during processing that requires effort (as opposed to automatic and unconscious processes). Note that, in tasks requiring metaphor comprehension, subjects with depression performed just as well as healthy subjects. Assuming that processing of metaphorical content is automatic and effortless, we find confirmation of the speculation that the basic level of information processing is not disturbed as a result of depression, in agreement with the predictions of Williams’ integrated theory (1988, 1997, as cited in Ellwart et al. 2003). It is also compatible with the concept of Sędek and associates (2010) explaining depressive cognitive deficits by referring to the cognitive exhaustion model (Kofta and Sędek 1998; Sędek and Kofta 1990; Sędek et al. 1993; reviewed in Sędek et al. 2010) and anticipating, among other things, that depression reduces the capacity for creating mental models of complex problems, but without perceptibly affecting the performance of tasks requiring automatic information processing. There are also other results of empirical studies (e.g., Ellwart et al. 2003) confirming the idea that cognitive problems in depressive subjects appear particularly when additional burdens are placed on the cognitive system.

#### Testing Hypothesis 1b

Contrary to Hypothesis 1a, Hypothesis 1b has been confirmed by the present results. Compared to healthy people, depressive subjects chose sentences about SADNESS that had a high level of metaphoricity as being more accurate than sentences that were more literal. A similar pattern of results was observed for the temporal notions PAST and FUTURE, in agreement with Hypothesis 1b if we assume that these notions have negative valence for people suffering from depression.

The obtained pattern of results can be interpreted in at least two ways. First of all, it can be treated as confirmation of one of the elements in Beck’s cognitive triad (1963, 1967). In this concept, the FUTURE, next to the WORLD and the SELF, is the topic area around which the automatic negative thoughts of patients with depression revolve. A greater number of metaphorical conceptualizations of the PAST (and FUTURE and SADNESS) could be evidence that people with depression are more concentrated on topic areas related to these notions than healthy people. This is in agreement with reports on the topics of depressive ruminations (cf. Joormann and Gotlib 2008), with the results of studies suggesting excessive self-absorption of depressive subjects (e.g., Huflejt-Łukasik 2010), and with theories that a depressive mood inclines individuals to process information that conforms to it (e.g., resource commitment theories; for a review, see Piotrowski and Wierzchoń 2009).

An alternative interpretation for the larger number of metaphorical conceptualizations of PAST, FUTURE, and SADNESS produced by the experimental group can be found in theories suggesting defocused attention in depressive subjects (see e.g., von Hecker and Meiser 2005). Let us assume that metaphorical understanding of notions causes activation of their less prototypical semantic qualities. This is in agreement with the results of studies suggesting that, compared to healthy people, subjects with depression more often notice qualities of a stimulus that are irrelevant for the cognitive task in hand (von Hecker and Meiser 2005). It needs noting, however, that if we accept this interpretation, the question arises as to why intensified metaphorical processing only occurred for selected notions and not all of them. Could this feature of cognitive function be activated only for stimuli that match a depressive mood? The results of studies carried out so far do not confirm such a correlation.

#### Testing Hypothesis 1c

Hypothesis 1c, which concerns inter-group differences in the valence of notion representation, was strongly and unequivocally confirmed by the present results (statistically significant inter-group differences for FUTURE, JOY, HAPPINESS, and SADNESS). This result can be regarded as an important contribution to the discussion on the nature of depressive cognition distortions. First of all, it turns out that the negative cognitive patterns suggested by Beck also manifest themselves at the notion level. Depression appears to be a negative interpretation filter placed over thought patterns, as well as processes of comprehending individual notions. Secondly, the obtained pattern of results could provide inspiration for expanding the scope of research on cognitive disturbances in depression. To the best of our knowledge, existing theoretical models have concentrated on distortions in processing stimuli with different affective characteristics typically associated with depression (cf. review by Bylsma et al. 2008, dividing theoretical proposals into concepts speaking of sensitivity to negative emotional stimuli, including the content-specificity hypothesis of Beck 1976, concepts focusing on difficulties with reacting to positive stimuli, and theories speaking of insensitivity to all emotional stimuli in depression). The present study takes a different look at the problem: It proposes investigating not so much the influence that the valence of the notion itself has on its ease of processing by a depression sufferer, but the interpretation distortions that emerge in depressive subjects’ processing of verbal stimuli of different valence.

One possible explanation for the interpretational negativism observed in the study’s depressive subjects could be the influence of ruminative thoughts on cognitive processes. It has been pointed out that they can be activated by a negative mood and persist for a greater length of time due to depressive subjects’ deficits in refreshing working memory, amongst other factors (Joormann and Gotlib 2008). It is possible that active negative representations, and difficulties with replacing them with positive ones, which would serve mood improvement, are conducive to negative processing of received stimuli, including verbal ones. Ruminations about one’s own depressive mood, symptoms, anxieties, and fears favor assimilation of stimuli in such a way as to make them compatible with the ruminations in terms of content. The results obtained in the present study would seem to confirm this. The literature includes research results compatible with this that concern processing of nonverbal stimuli (cf. e.g., the study of Gollan et al. 2008, on identifying facial expressions).

#### Testing Hypothesis 2

Hypothesis 2 concerned the persistence of cognitive changes during remission from depression. In view of the results of many recent studies (e.g., Atchley et al. 2007; Holmes and Pizzagalli 2007; Watkins et al. 2000: bias in conceptual processing), the pattern of results for people during remission from depression was expected to be similar in many respects to those of depressive subjects, even if the typical qualities were less prominent. Contrary to expectations, people during remission from depression (R) not only did not give similar replies to subjects with depression (E), but their results were also strikingly similar to those of the non-depressive control group (C): For four out of the five notions studied (PAST, FUTURE, SADNESS, HAPPINESS), it even turned out that four items chosen as being the most accurate were the same in both groups. All the observed statistically significant differences between groups E and R also occurred between groups E and C, and concerned exactly the same aspects and the same notions.

Interpreting the results, they do not seem to show the existence of depression-related cognitive distortions in notion comprehension in the group of subjects during remission from depression. This result contradicts conclusions suggesting that cognitive changes persist in people who previously had a depressive episode. In view of inconsistent study results described in the literature, it is hard to draw any firm conclusions. The first interpretation that comes to mind is that cognitive changes simply recede during remission. Such results have been reported before (e.g., Barnett and Gotlib 1988; as cited in Ilardi and Craighead 1999). In fact, subjects in remission did reply very similarly to the control group. Furthermore, taking valence into account, in a few cases their replies were even more “non-depressive” (higher valence) than the replies of healthy subjects. This opens up interesting possibilities of interpretation: Could a depressive episode in the past act as a kind of vaccine, “redirecting” conceptual mechanisms to non-depressive paths (e.g., by activating positively marked semantic networks)? Such an explanation does not dispel all doubts, however, e.g., how would we explain a tendency for depression to recur in the future, observed in some patients with a depressive episode in their case history?

Let us consider a different possibility. One could assume that depression-related cognitive changes persist (though with reduced intensity) during remission of depression, but not on the notion comprehension level. This interpretation is exceptionally inspiring, too. Could we assume, for example, that disturbances at the notion level are typical of full-blown depression? Verification of this supposition would open up some promising diagnostic possibilities. However, for valid conclusions to be drawn, more research is definitely needed.

### Limitations of the Study Results

The limitations of the study results are related in particular to the pharmacotherapy of depressive patients and the way in which the group of patients in remission was selected. As a reminder, members of the experimental group were chosen from among patients of psychiatric wards and outpatient clinics. For obvious reasons, it would have been unethical to interrupt their pharmacotherapy for the period of the study. It is important to remember that patients suffering from depression were taking antidepressants. These drugs alleviate the symptoms of depression, and there is also evidence of their beneficial influence on cognitive processes (see Talarowska et al. 2009). Therefore we can say with substantial probability that the drugs the depressive patients were taking could have affected their performance in the tasks. Perhaps a comparison of the results of healthy subjects and subjects with untreated depression would have shown greater inter-group differences.

Another major limitation to a generalization of the results is the specificity of the group of patients during remission of depression (R). When the subjects were being selected for the study, the conditions for including a person in the group were (a) having had a depressive episode in the past and (b) the current remission of depression, that is, the lack of depressive symptoms during the study. The indicator of depressive symptoms was, in addition to a medical diagnosis, the Beck Depression Inventory result. Similarly to other studies involving depressive subjects (Beck et al. 1987; Ruscio and Ruscio 2002; see also Fajkowska and Marszał-Wiśniewska 2006), it was accepted that an individual was not currently suffering from depression if their result was less than 10. This condition made it much harder to find members for group R. Most of the patients seeing their doctors for checkups, whose case histories read “recurrent depressive disorder, currently in remission,” achieved BDI results suggesting a depressive state. In the study’s several months, working with four psychiatrists, only 12 subjects were found who met the criteria. The question of whether the sample was representative, whether the members of group R were typical during remission from depression, remains unanswered. The present study failed to confirm the hypothesis regarding the persistence of depression-related cognitive distortions in group R. It needs noting, however, that its members were a precisely selected group, so any generalization of this result should be offered with caution.

Apart from the pharmacotherapy of depressive patients, and the way in which the group of patients in remission was selected, two more additional factors have to be considered: the sex disparity and the broad age range of the participants in the study. As regards the sex disparity, it needs noting that in all three groups of participants, women significantly outnumbered men: In Groups E and C, there were 80 % females ($n = 24$), and in Group R, 67 % females ($n = 9$, see Table 1). This could result from the fact that depression is more frequent among women (cf. Bilikiewicz et al. 2002, Vol. 2, p. 387), but it could also be that women were simply more numerously represented among the patients of the four psychiatrists that cooperated with us in the study. Either way, when discussing our results, the sex disparity may be taken as a limitation of the study results, all the more since there is a lot of evidence that women and men differ in their processing of language (cf. e.g., Pycia 2011: the case of Polish and Croatian languages).

Regarding age, both twenty-year-olds and elderly people participated in our project. We carefully selected the participants so as to ensure that each “decade” (20–29, 30–39, 40–49 years, etc.) was equally represented in each group. We also made all three groups comparable with regard to the age of the participants. Nevertheless, age could be confusing. For instance, if age was controlled for, we would probably obtain a different pattern of results (e.g., elderly people could perform worse, not because of depression, but due to age-related retardation, i.e., a general slowing down of mental operations observed in the elderly population; see e.g., Brzezicka-Rotkiewicz and Sędek 2005).

### Research Tasks for the Future

In particular, we now discuss plans for research on the cognitive representations of other notions, projects investigating working memory efficiency and intensity of ruminative thoughts, and work on tools for differentiating between conceptual processing in healthy people and in those during remission from depression.

When planning research projects inspired by the present results, the first thing that springs to mind is expanding the research area to include other notions important from the point of view of symptoms of depression, for instance SELF, WORLD, the PRESENT, DANGER, FATIGUE. Considering how fruitful the study on representations of the PAST, FUTURE, JOY, SADNESS, and HAPPINESS has been, one can presume that studies on comprehension of other notions would also yield interesting results.

It would also seem advisable to extend the tools to include a task checking the capacity of the subjects’ working memory (e.g., an $n$-back task). In our study, we decided not to include any task measuring working memory span because of the specificity of the group being studied. People suffering from depression become tired very fast and unwillingly decide to perform any cognitive tasks. We had severe problems with encouraging depressive patients to take part in our study, and many of them withdrew after they saw the questionnaire they would have to fill in. After a pilot study, we had to simplify and shorten our materials. That is why we decided not to include any additional tasks and to rely on the broad literature confirming the existence of the working memory deficit in depression. Our results led to the conclusion that working memory deficits related to depression do not impair the process of metaphor comprehension, which is automatic and effortless. However, further research is needed to confirm this idea, such as studies investigating working memory capacity and taking into account a larger number of diverse stimuli. For similar reasons, it would be worth including a tool measuring the intensity of ruminative thoughts in the studied subjects (cf. e.g., *Ruminative Response Scale of the Response Style Questionnaire*; Nolen-Hoeksema and Morrow 1991, cited in Levens et al. 2009). An interesting idea suggested by the analysis of results of subjects during remission of depression could be to consider the number of depressive episodes they experienced in the past, and the significance of this variable’s influence on the obtained results. Talarowska et al. (2009) refer to the number of earlier depressive episodes as one of the factors influencing an individual’s cognitive function.

Another extremely interesting task for the future could be to develop a tool sensitive to any differences in the conceptualization of notions by persons during remission from depression, and persons without any depressive episodes in their case history. Such differences were identified in one study conducted by our team (for a description of the method and results, see Bartczak 2011b; Bartczak and Bokus 2013). The task did not involve creating metaphorical and non-metaphorical conceptualizations with different valence, but assigning semantic distances between the notions being studied (for an example of the use of a different semantic distance latency test, see e.g., Chiao et al. 2004). The subjects were asked to treat the notions like guests who had come to a party and to seat them (together with themselves and two other “guests”—notions) at a round table. Based on the way the “guests” were placed around the table, distances between the notions were assigned numerical values. This was done as follows: The distances represented by guests X and Y sitting next to each other were given a value of 1. If guests X and Y were separated by one “person” the value was 2, if two “people”—3, and if they were separated by three “people”—4. The results obtained using this tool differentiated between all three groups of subjects, including patients during remission from depression (see Bartczak 2011b; Bartczak and Bokus 2013). Considering the simplicity of these kinds of tools and their easy application, they seem valuable tools to use (after some extra studies and perhaps improvements) for diagnostic purposes, e.g., as one of the methods monitoring the progress of therapy. It is also interesting to consider work on the Semantic Distance Taks as a simple tool for distinguishing between groups of subjects who are depressive, healthy, and in remission. Moreover, building a tool leading to results in the form of a complex data set allows the ultimate analysis to be based on the increasingly popular data mining method.

## Appendix

See Appendix Tables 2 and  3.

## Footnotes

1 F32.1—moderate depressive episode. F33.1—recurrent depressive disorder, current episode moderate.

2 F33.4—recurrent depressive disorder, currently in remission. F32—depressive episode. The research tools also included the Unfinished Metaphorical Sentences Test, a test of association (cf. Bartczak et al. 2010) and the Semantic Distance Task (see Bartczak and Bokus 2013). For a presentation of the results obtained using these tools, see Bartczak 2011b.

3 The research tools also included the Unfinished Metaphorical Sentences Test, a test of association (cf. Bartczak et al. 2010) and the Semantic Distance Task (Bartczak and Bokus 2013). For a presentation of the results obtained using these tools, see Bartczak (2011b).
